# The effects of interventional mitral valve repair using the MitraClip System on the results of pulmonary function testing, pulmonary pressure and diffusing capacity of the lung

**DOI:** 10.1186/s12872-021-02042-1

**Published:** 2021-05-11

**Authors:** Lucie Kretzler, Stephan Große, Stephan Wiedemann, Carsten Wunderlich, Chris Nowak, Christian Riedel, Tomáš Sieger, Steffen Schoen

**Affiliations:** 1 Clinical Study Center (CSC), Berlin Institute of Health at Charité – Universitätsmedizin Berlin, Charitéplatz 1, 10117 Berlin, Germany; 2 Department of Internal Medicine and Cardiology, Pirna Hospital, Pirna, Germany; 3 Department of Cybernetics, Faculty of Electrical Engineering, Czech Technical University in Prague, Prague, Czech Republic

**Keywords:** MitraClip, Pulmonary function, Patient registry, Valvular disease, Structural intervention

## Abstract

**Background:**

The study analyzes changes in lung function, pulmonary pressure and diffusing capacity of the lung in patients with mitral valve regurgitation (MR) treated by MitraClip implantation.

**Methods:**

A total of 43 patients (19 women and 24 men with an average age of 78.0 ± 6.6 years) who were able to perform pulmonary function testing including diffusing capacity of the lung for carbon monoxide (DLCO), vital capacity (VC), total lung capacity (TLC), residual volume (RV) and forced expiratory volume in 1 s (FEV1) before and 6 weeks after MitraClip implantation participated in this study. Furthermore, clinical and echocardiographic parameters including systolic pulmonary artery pressure (sPAP), left ventricular ejection fraction (LVEF) and left atrial diameter (LAD) measurements were recorded in all patients.

**Results:**

The procedure was performed successfully in all 43 patients leading to a reduction of MR in 97.7% of cases. One patient died on day 4 after the intervention most likely due to pulmonary artery embolism. Six weeks after the implantation 79.1% of patients showed a MR of at most mild to moderate. Furthermore, we could demonstrate a significant reduction of systolic pulmonary artery pressure during follow-up (from 48.8 ± 11.4 mmHg to 42.9 ± 9.0 mmHg (t(41) = − 2.6, p = 0.01). However, no changes in LVEF were detected. Comparing pre and post implant lung function tests, no significant alterations were seen for VC, TLC, DLCO and FEV1. Though, in a subgroup of patients with moderate to severe preexisting deterioration of DLCO at the baseline (max. 50%) the MitraClip procedure resulted in a significant improvement in DLCO (37.8% ± 9.0 to 41.6% ± 10.0, p < 0.001).

**Conclusions:**

Treatment of MR with the MitraClip system successfully reduces MR severity in the vast majority of patients. Consecutively, a reduction in pulmonary pressure could be observed, however no changes in LVEF were obvious. Lung function tests remained unaltered during follow-up. However, in a subgroup of patients with severe preexisting deterioration of DLCO the MitraClip procedure resulted in a significant improvement in DLCO.

**Trial registration:**

Name of the registry: Die Auswirkung der interventionellen Mitralklappenreparatur mit MitraClip-System auf die Ergebnisse der Lungenfunktionsmessung.

**Trial registration number:**

DRKS00022435; Date of registration: 09/07/2020 'Retrospectively registered'; URL of trial registry record: https://www.drks.de/drks_web/navigate.do?navigationId=trial.HTML&TRIAL_ID=DRKS00022435.

## Background

Mitral valve regurgitation (MR), the most common type of valvular heart disease, affects nearly 10% of people above the age of 75 years [1]. It is—aside from aortic valve stenosis—the second most frequent indication for heart valve surgery in Europe [2]. MR is classified as primary, when the underlying pathology includes a structural or degenerative abnormality of the mitral valve itself. The secondary MR is caused by left ventricular dysfunction. Secondary MR can develop due to both ischemic and nonischemic cardiomyopathies, in which the pathological processes of remodeling of the ventricle or the atrium lead to a consecutive insufficiency of the mitral valve apparatus comprising leaflets, chordae tendineae, papillary muscles, or mitral annulus. In chronic MR, there is no drug proven to improve long-term outcomes [3], whereas surgery and transcatheter mitral valve repair show positive effects [4]. The MitraClip is a polyester-covered cobalt-chromium device, which can be implanted percutaneously through the femoral vein into the left atrium following transseptal puncture [5]. The clipping procedures based on the surgical technique first described by Alfieri. This intervention receives a IIb recommendation in the recent AHA/ACC valve guideline update for patients with NYHA class III-IV symptoms in a setting of chronic severe MR despite optimal medical treatment with favorable mitral valve anatomy, a reasonable life expectancy, and a high risk for mitral valve surgery [3]. Due to reduction of the mitral valve regurgitation volume after MitraClip implantation the procedure has been associated with acute reduction in pulmonary artery pressure (PAP) evaluated by echocardiography or invasively by right heart catheterization (RHC) in previous studies [6]. Right heart catheterization is considered the gold standard, but it remains a time-intensive and invasive technique that may preclude its use as a frequent method of follow-up in patients with pulmonary hypertension. Consequently, diffusing capacity of the lung (DLCO)—a non-invasive technique of CO assessment—may be a good agreement compared to thermodilution and the Fick method in RHC examinations, which can be simply conducted in most patients [7]. Increased left atrial pressure leads to the elevation in pulmonary venous pressure, which is in turn transferred to pulmonary capillaries causing damage to the alveolar-capillary barrier [8]. We hypothesize, that this mechanism leads to the decrease of DLCO, which should improve after the reveal from the valvular disease following the clip procedure. Despite rapidly increasing numbers of interventional mitral valve repairs, these alterations are still assumptions, and up to now little is known about the effects of the treatment on pulmonary circulation and changes in lung function in these patients.

## Scientific aims and perspectives

The study analyzes changes in diffusing capacity of the lung for carbon monoxide (DLCO), vital capacity (VC), total lung capacity (TLC), residual volume (RV) and forced expiratory volume in 1 s (FEV1) in patients with MR before and 6 weeks after MitraClip implantation. Furthermore, clinical and echocardiographic parameters including systolic pulmonary artery pressure (sPAP) and left ventricular ejection fraction (LVEF) were done to detect changes in hemodynamic parameters.

## Methods

This observational, prospective, single-arm, single center clinical study was designed to compile real-world clinical outcome data for percutaneous edge-to-edge mitral valve repair technology utilizing the MitraClip device (Abbott, Menlo Park, CA, USA). The registry was initiated in May 2016. This study was approved by the University of Dresden (Germany) and was performed at the Cardiology Department of HELIOS Hospital Pirna.

### Patient selection

All participants had to have the following characteristics (inclusion criteria): (A) Men and women of any ethnicity aged at least 18 years; B) Indication for interventional mitral valve repair using the MitraClip device according to the current available evidence regarding this treatment; (C) Ability to give an informed consent after oral and written information. For the present study, additional exclusion criteria were: A) Simultaneous participation in other studies; B) Lack of informed consent to the evaluation of their personal and medical data. One day before the procedure, anamnestic details, clinical examination, blood collection, echocardiography, spirometry, and body plethysmography with diffusion measurement were performed. After presentation to the heart-team, consisting of cardiac surgeons as well as interventional cardiologists, and a positive vote, patients were screened for participation in an all-comers design between May 2016 and March 2019. The echocardiographic assessment of MR severity was as follows: none, mild, moderate and severe. Written informed consent, was obtained from all participants. All patients treated with MitraClip between 2016 and 2019 have been considered in the study.

### Procedural details

43 patients were able to perform pulmonary function testing including diffusing capacity of the lung for carbon monoxide (DLCO), vital capacity (VC), total lung capacity (TLC), residual volume (RV) and forced expiratory volume in 1 s (FEV1) before and 6 weeks after MitraClip implantation. Furthermore, clinical and echocardiographic parameters including systolic pulmonary artery pressure (sPAP), left ventricular ejection fraction (LVEF) and left atrial diameter (LAD) measurements were recorded in all patients.

The pulmonary function parameters were measured using the single-breath method with the Master-Screen™pneumo spirometer (CareFusion Corporation, San Diego, CA, USA) by experienced medical-technical assistants for functional pulmonary diagnostics after standardized pre-test training maneuvers. The measurements of single breath diffusing capacity or transfer factor of the lung for carbon monoxide (DLCO), DLCO related to the alveolar volume (DLCO/VA), vital capacity (VC), total lung capacity (TLC) and forced expiratory volume in 1 s (FEV1) were conducted before and 6 weeks after the MitraClip procedure.

Patients underwent echocardiographic imaging with a GE VIVID E95 (General Electric Company, Boston, Massachusetts, USA) by an experienced cardiologist. Left ventricular ejection fraction was calculated using the Simpson method. Measurements were done before and 6 weeks after MitraClip implantation.

The MitraClip procedure was carried out in the cardiac catheter laboratory (Philips, Allura Xper FD 10) under fluoroscopy and 3D-TEE guidance according to the instructions for use under general anesthesia.

After the procedure, the patients were monitored in the intensive care unit for 24 h. Before discharge, a transthoracic echocardiography was performed to control the short-term outcome after intervention.

### Statistical analysis

The data were collected and analyzed in Microsoft Excel 2010 database (Microsoft Corporation) and R version 3.2.4 (The R Foundation for Statistical Computing) in combination with R Studio were used for the statistical work and for creating the diagrams and graphics. Quantitative data are expressed as mean ± SD. Categorical data are given in proportions and percentages. Statistical comparisons of quantitative data were performed with a 2-tailed t-test for paired samples in continuous data containing no highly outlying values. The Wilcoxon exact test was used in continuous data with outliers as a more powerful alternative to the t-test. The binomial test was used to assess the significance of the number of patients whose DLCO improved after the procedure. Linear model was used to compare the improvement in DLCO, and sPAP, respectively, between subgroups of patients. A value of *p* < 0.05 was considered statistically significant.

## Results

### Analysis of the basic patients’ characteristics, comorbidities and estimated surgical risk

A total of 19 women with an average age of 76.5 ± 8.0 years (median 79 years) and 24 men with an average age of 79.2 ± 5.1 years (median 80 years) participated in the study. The echocardiographic measurements as well as measurements of DLCO, VC, TLC, RV and FEV1 before and after the procedure were conducted in 42 patients, one patient died on day 4 after the intervention most likely due to pulmonary artery embolism. Comorbidities and baseline characteristics of survived patients are displayed in Table 1. None of the patients was on renal replacement therapy. Neither had any patient long-term oxygen therapy.

**Table 1:** Baseline characteristics of patients. Mean values ± standard deviations are given

Age (years)	77.6 ± 6.6
Weight (kg)	79.1 ± 14.7
BMI (kg/m2)	27.0 ± 4.9
Female gender, n (%)	18/42(42.9%)
NYHA functional class, n (%)	II 1/42(2.4%)
	III 40/42 (95.2%)
	IV 1/42 (2.4%)
Etiology of the MR, n (%)	(1) Primary 23/42(54.8%)
	(2) Secondary 17/42(40.5%)
	(3) Mixed 2/42(4.8%)
Chronic obstructive lung disease	7/42 (16.7%)
Asthma bronchiale	1/42(2.4%)
LVEF (%)	48.2 ± 13.4
N-terminal pro-B-type natriuretic peptide (pg/ml)	3367.7 ± 4718.0
EURO Score II	5.0 ± 3.6
Pulmonary artery pressure (mmHg)	48.8 ± 11.4
Glomerular filtration rate (mL/min/1.73 m2)	53.2 ± 22.2
Vital capacity (ml/kg)	85.3 ± 21.8
Left atrial diameter in M-Mode (mm)	48.1 ± 8.0
Atrial fibrillation (AF), n (%)	35 (83.3%)

### Procedural success

The implantation of one or more clips was successfully performed in all patients. In 34 patients (79.1%) the reduction of the mitral valve insufficiency was considered sufficient, defined as a residual MR of at most mild. No clip embolization and no pericardial tamponade occurred during the duration of the study. 17 (40.5%) patients received 1 clip, 14 (38.1%) patients received 2 clips and 5 patients (11.9%) received 3 clips.

### The impact of the MitraClip procedure on pulmonary function test results and echocardiographic parameters

**Fig. 1:**
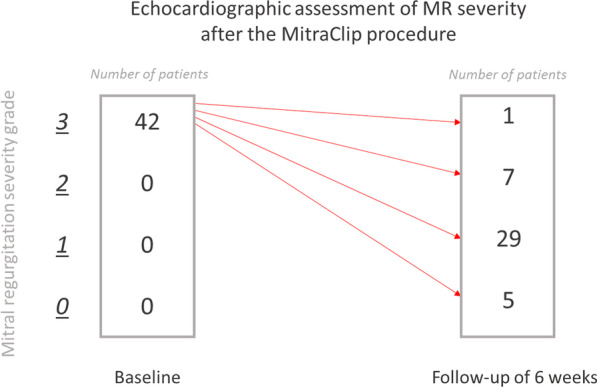
Echocardiographic assessment of MR severity before and after the MitraClip procedure

Follow-up 6 weeks after implantation (53.0±21.9 days) echocardiography revealed reduction of MR to at least moderate in 97.7% of cases. Figure 1 displays severity of MR before and after MitraClip in 42 patients.

**Fig. 2:**
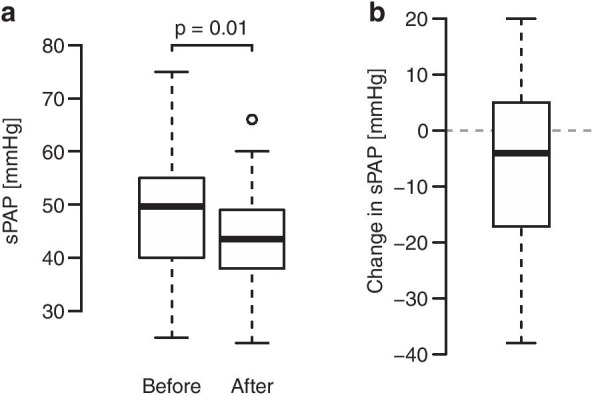
Echocardiographic assessment of pulmonary artery pressure (mmHg) before and after the MitraClip procedure. A: The systolic pulmonary artery pressure decreased from 48.8 ± 11.4 mmHg to 42.9 ± 9.0 mmHg, p = 0.01). B: The differences in systolic pulmonary artery pressure. sPAP = pulmonary artery pressure

At 6 weeks after the procedure patients exhibited a significant reduction in systolic pulmonary artery pressure from 48.8 ± 11.4 mmHg to 42.9 ± 9.0 mmHg (t(41) = − 2.6, *p* = 0.01) (Fig. 2).

**Fig. 3:**
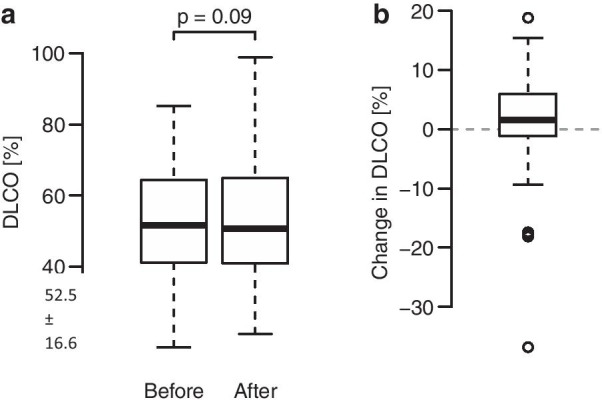
Diffusing capacity of the lung for carbon monoxide. A: The diffusing capacity did not differ before and after the MitraClip procedure, even though it increased in most patients. B: The differences in the diffusing capacity. DLCO = Diffusing capacity of the lung for carbon monoxide

The increase in DLCO values after the MitraClip implantation was not significant (increase from 52.5 ± 16.6% to 53.1 ± 17.8%; Wilcoxon exact test *p* = 0.09) in the whole study cohort, but the number of 28/42 (67%) patients in whom DLCO increased after implantation was significantly higher than expected by chance (binomial test *p* = 0.04) (Fig. 3).

**Fig. 4:**
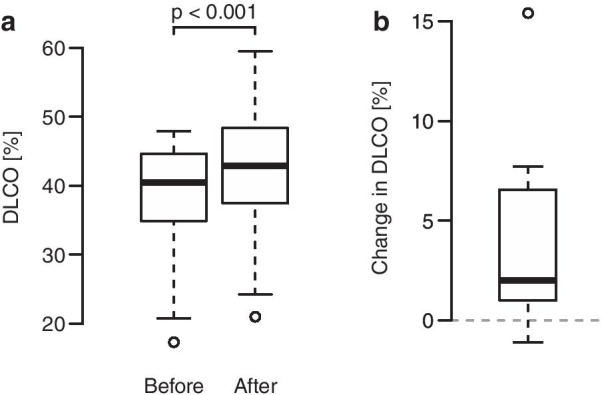
Diffusing capacity of the lung for carbon monoxide in patients with moderate to severe deterioration of DLCO-values at the baseline (max. 50%). **a** The diffusing capacity increased after the MitraClip procedure in the patient subgroup. B The differences in the diffusing capacity. DLCO = Diffusing capacity of the lung for carbon monoxide

Moreover, considering the subgroup of 19 patients with moderate to severe preexisting deterioration of DLCO at the baseline (max. 50%) the MitraClip procedure resulted in a significant improvement in DLCO (37.8 ± 9.0% to 41.6 ± 10.0%) in the follow-up using a Wilcoxon exact test (*p* < 0.001). Seventeen out of 19 patients in the subgroup improved after the implantation (binomial test *p* < 0.001) (Fig. 4).

**Fig. 5:**
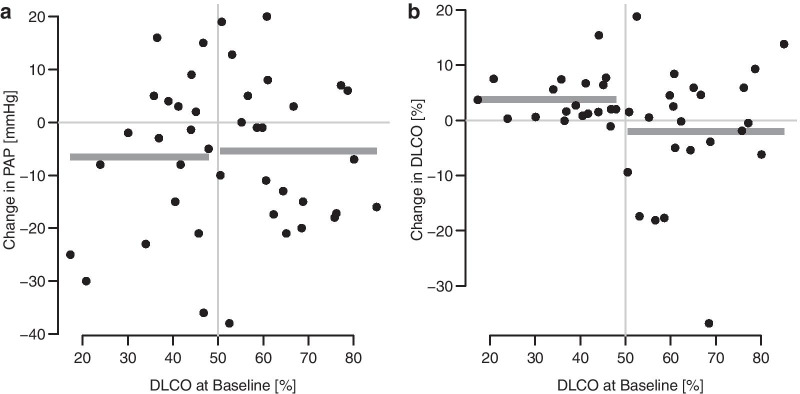
Improvement in pulmonary artery pressure and diffusing lung capacity after the MitraClip procedure. **a** The pulmonary artery pressure decreased in patients with DLCO <  = 50% as well as in patients with DLCO > 50%. **b** The diffusing capacity of the lung for carbon monoxide increased in patients with DLCO <  = 50%, but not in the other patients. The gray horizontal bars denote mean values in subgroups. sPAP = Systolic pulmonary artery pressure, DLCO = Diffusing capacity of the lung for carbon monoxide

We considered in greater depth the improvement in sPAP and DLCO, respectively, in the subgroup with DLCO <  = 50% vs. in the rest of patients (Fig. 5, Table 1). The values of sPAP decreased by 6.5 mmHg in the diseased subgroup (t(18) = − 1.9, *p* = 0.07), and by 5.4 mmHg in the rest of patients (t(22) = − 1.8, *p* = 0.08). The difference of those effects was not significant (t(40) = − 1.1, *p* = 0.81, Fig. 5a). However, the values of DLCO increased by 3.8 in the diseased subgroup (t(18) = 4.1, p < 0.001), and by 2.0 in the rest of patients (t(22) = − 0.8, *p* = 0.43). The difference in those effects was of borderline significance (t(40) = 5.8, *p* = 0.052, Fig. 5b). Three patients transitioned to more than 50% of DLCO from less than 50%. Three patients improved by at least 10%. In 7 patients with COPD, the DLCO insignificantly decreased postoperatively by − 3.1 mmHg (t(6) = 1.0, *p* = 0.38, the number of decreases = 4). In 34 patients without COPD, asthma, or ACOS, the DLCO insignificantly increased by 1.9 mmHg (t(33) = 1.2, p = 0.25, the number of increases = 25).

**Fig. 6:**
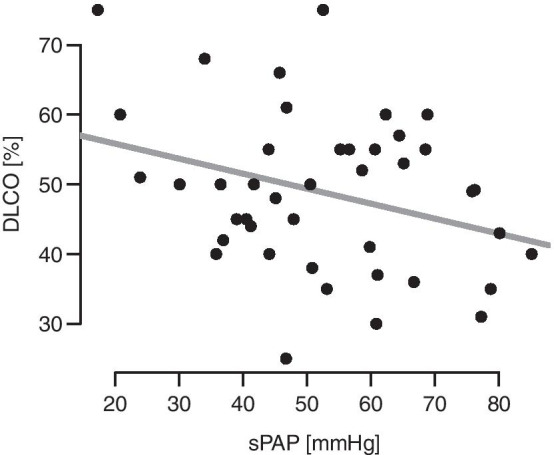
Diffusing capacity of the lung for carbon monoxide correlated with pulmonary artery pressure *(p* = 0.04). sPAP = Pulmonary artery pressure, DLCO = Diffusing capacity of the lung for carbon monoxide

The values of sPAP correlated with DLCO (t(40) = − 2.1, *p* = 0.04)(Fig. 6).

Our cohort had a mean RV of 130.0 ± 31.9% before intervention with a statistically significant reduction 119.4 ± 29.3% (*p* = 0.007) at follow-up (Table 2). The residual volume does not participate in gas exchange, so a reduction corresponds to clinical improvement.

**Table 2:** Estimates of DLCO, VC, TLC, RV and FEV1 in patients with primary and secondary mitral regurgitation before and after the MitraClip procedure. *p* values are based on Student’s *t* test with the exception of DLCO, for which it is based on the Wilcoxon exact test due to outlying data values

	Before intervention	After intervention	Difference	*p* value
Mean DLCO (%)	52.5 ± 16.6	53.1 ± 17.8	0.6 ± 9.7	0.09
Mean VC (ml)	85.3 ± 21.8	86.2 ± 19.0	0.9 ± 13.9	0.67
Mean TLC (ml)	99.2 ± 18.5	95.8 ± 16.2	− 3.3 ± 12.7	0.10
Mean RV (ml)	130.0 ± 31.9	119.4 ± 29.3	− 10.3 ± 23.2	0.007
Mean FEV1 (ml)	81.0 ± 24.5	80.8 ± 24.7	− 0.3 ± 12.8	0.89

## Discussion

The aim of the study was to analyze changes in lung function tests and echocardiographic parameters before and 6 weeks after MitraClip implantation in patients with MR. The MitraClip procedure could be performed successfully in all 43 patients leading to a reduction of MR in 97.7% of cases. However, one patient died on day 4 after the intervention most likely due to pulmonary artery embolism. 42 patients were analyzed before and 6 weeks after implantation. 79.1% of them showed an MR of at most mild. Furthermore, we could demonstrate a significant reduction of pulmonary artery pressure during follow-up. No changes in LVEF were detected. These data are in good accordance with data from the COAPT trial [4]. We haven´t performed six-minute walk test (6MWT) in this study because changes of 6MWT, showing an improvement of the walking distance after MitraClip have been published before [13]. Comparing pre and post implant lung function tests, no significant alterations were seen for VC, TLC, and FEV1. The values of DLCO improved in most patients and in a subgroup of patients with severe preexisting deterioration of DLCO the MitraClip procedure resulted in a significant improvement in DLCO. These findings demonstrate a reversibility of pulmonary dysfunction due to MR, as this might be attributed predominantly to interstitial and alveolar edema. The quantitative evaluation of the pulmonary diffusing capacity reflects the state of the pulmonary interstitium and the alveoli. As soon as the diffusion process in the lung is impaired, hypoxemia and dyspnea occur [9]. According to Zou et al. the value of DLCO can be used as a non-invasive screening tool for the identification of exercise PH in a referral population [12]. DLCO measures the transfer of inhaled gas to red blood cells within pulmonary capillaries and reflects the properties of the alveolar–capillary membrane. Reduced DLCO in pulmonary arterial hypertension (PAH) may be the result of vascular remodeling being associated with a proportional reduction in the diffusion capacity of the alveolar capillary membrane and the total pulmonary capillary blood volume available for gas exchange [13]. Evidence suggests, that patients with long-term mitral valve disease may also present with bronchial hyperreactivity [10]. From a pathophysiologic point of view, the reduction of sPAP could be associated with the reduction of the compression of the small airways. FEV 25–75%, which reflects changes exclusively in the small airways, should thus be considered for analysis in the future trials. The present results indicate an improvement of DLCO after MitraClip implantation in patients with MR even if the preexisting DLCO is severely deteriorated. According to Mustafa et al., deterioration in pulmonary function is correlated to severity of cardiac dysfunction and may be reversible late after valve surgery, except in very advanced cases that have poor prognosis [11]. In this study, cardio surgery patients with severe valve disease who started with low values of DLCO showed improvement in the early postoperative period. We attribute this result to the reduction in alveolar and interstitial fluid amount due to improvement of the heart failure. Thus, at least for the group of patients with moderately to severely impaired baseline diffusion capacity (DLCO ≤ 50%), our hypothesis was confirmed.

### Limitations

The small patient cohort, the focus on patients who were able to perform pulmonary function testing, the short follow-up and the lack of randomization with a control group constitute the natural limitations of this study. The bias represents a fact, that only a part of the whole MitraClip patient cohort treated in our center was available for the follow up measurements.

## Conclusion

Treatment of MR with the MitraClip system successfully reduces MR severity in the vast majority of patients. Consecutively a reduction in pulmonary pressure could be observed, however no changes in LVEF were obvious. Lung function tests remained mostly unaltered during follow-up, but DLCO improved in majority of patients. Moreover, in a subgroup of patients with severe preexisting deterioration of DLCO the MitraClip procedure resulted in a significant improvement in DLCO. Larger prospective trials are needed to confirm whether these effects translate into clinical outcomes.

## Data Availability

The datasets generated and analysed during the current study are available upon reasonable request from the corresponding authors.

## Abbreviations

MR: Mitral valve regurgitation
CO: Carbon monoxide
DLCO: Diffusing capacity of the lung for carbon monoxide
VC: Vital capacity
TLC: Total lung capacity
RV: Residual volume
FEV1: Forced expiratory volume in 1 s
sPAP: Systolic Pulmonary artery pressure
LVEF: Left ventricular ejection fraction
LAD: Left atrial diameter
RHC: Right heart catheterization
6MWT: Six-minute walk test
